# SWI/SNF chromatin remodelers in prostate cancer progression

**DOI:** 10.3389/freae.2023.1337345

**Published:** 2024-01-04

**Authors:** Sandra C. Ordonez-Rubiano, Brayden P. Strohmier, Surbhi Sood, Emily C. Dykhuizen

**Affiliations:** ^1^ Department of Medicinal Chemistry and Molecular Pharmacology, Purdue University, West Lafayette, IN, United States; ^2^ Interdisciplinary Life Sciences-PULSe, Purdue University, West Lafayette, IN, United States; ^3^ Purdue Institute for Cancer Research, Purdue University, West Lafayette, IN, United States

**Keywords:** SWI/SNF, BAF complex, prostate cancer, androgen receptor, neuroendocrine differentiation, tumor microenvironment, chromatin remodeling

## Abstract

Prostate cancer (PCa) is the most commonly diagnosed cancer and the second most common cause of cancer-related deaths in men in the US. The majority of PCa cases arise in the luminal cells of the prostate and develop into adenocarcinoma. Primary PCas are heterogeneous and have alterations in a variety of tumor suppressors and oncogenes; however, the vast majority are dependent on gene expression regulation by androgen receptor (AR), making it the focus for most targeted therapy development. As the incidence of PCa cases resistant to AR-targeted therapies rises, there is renewed attention on how additional genetic and epigenetic alterations contribute to PCa progression and resistance. In this review we summarize the efforts made over the past 20 years to dissect the function of the SWI/SNF chromatin remodelers in PCa. We mainly focus on how SWI/SNF complexes regulate different aspects of AR signaling, facilitate other key drivers in PCa, promote the advancement of the disease, and regulate the tumor microenvironment.

## 1 Introduction

Prostate cancer (PCa) is a highly prevalent malignancy with 1.4 million cases diagnosed worldwide in 2020 alone (Sung et al., 2021). Approximately 288,000 new cases and 34,700 PCa-associated deaths are estimated for 2023 in the US (Siegel et al., 2023). PCa initiates as prostatic intraepithelial neoplasia, wherein malignant cells grow toward the lumen of the prostate (Wang et al., 2018). If the cancer advances, most PCa cases develop into prostate adenocarcinoma, which originates from luminal or basal cells in the prostate (Wang et al., 2018). PCa is a hormone-driven malignancy; therefore, the majority of the available therapies focus on disrupting hormone-related pathways (Wang et al., 2018; Teo et al., 2019; Litwin and Tan, 2017). Uncommon types of PCa include squamous carcinoma, which is derived uniquely from basal cells, and neuroendocrine carcinoma, which may arise from prostate neuroendocrine cells or as a result of resistance to treatment (Wang et al., 2018). As the malignancy progresses to metastatic disease, bone metastases are the most common, followed by lung and liver (Wang et al., 2018) (Figure 1).

**FIGURE 1:**
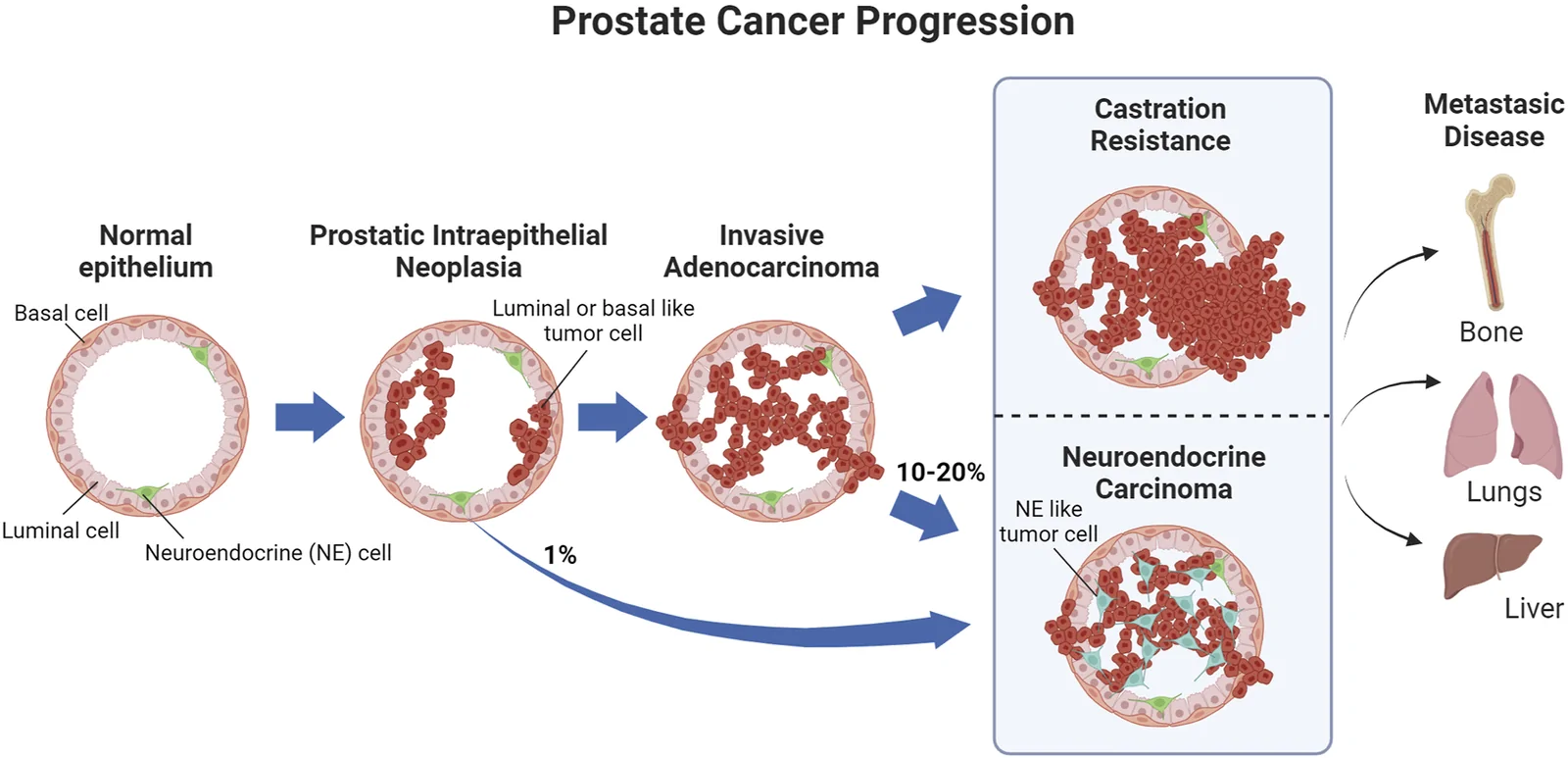
PCa progression. Prostatic intraepithelial neoplasias primarily develop into invasive carcinomas, while approximately 1% develop into neuroendocrine carcinoma. As the invasive carcinoma advances and stops responding to treatment, it becomes castration resistant, with 10–20% of castration-resistant tumors emerging through neuroendocrine differentiation. Metastatic PCa, primarily in the bone, can occur before or after castration resistance.

The Gleason scoring system is the international standard to score prostatic carcinomas from tissue biopsies (Gleason and Mellinger, 1974; Litwin and Tan, 2017). Combined with the evaluation of serum prostate-specific antigen (PSA) levels, the stage and grade of the cancer, the Gleason score is used to assign PCa patients to one of 5 risk groups: very low, low, intermediate, high or very high risk (Mohler et al., 2016). The therapeutic approach to very low risk patients is active surveillance and surgical removal of localized carcinoma (Wang et al., 2018; Litwin and Tan, 2017). Most therapies for higher risk groups are based on androgen deprivation therapy (ADT), which generally includes chemical castration with the adjunct use of androgen receptor (AR) antagonists. Other common treatments include radical prostatectomy, chemotherapy, immunotherapy, and radiotherapy (Wang et al., 2018; Desai, McManus, and Sharifi, 2021). Resistance to ADT leads to castration-resistant PCa (CRPC), which may emerge either before or after the metastatic stage (Wang et al., 2018; Desai et al., 2021). Cell line models representing the different stages of PCa disease and responsiveness to hormones commonly used in pre-clinical research are summarized in Table 1.

**TABLE 1:** Summary of PCa cell-based models mentioned in this review.

Cell line	Type	AR expression	References
RWPE-1	Non-cancerous prostate epithelium	High	Bello et al. (1997), Saranyutanon et al. (2020)
LNCaP	Castration-sensitive PCa	High	Horoszewicz et al. (1980), Bokhoven et al. (2003), Saranyutanon et al. (2020)
R1-AD1	Castration-sensitive PCa	High	Li et al. (2013), Nyquist et al. (2013)
C4-2/C4-2B	CRPC	High	Thalmann et al. (1994), Saranyutanon et al. (2020)
DU145	CRPC	Low	Stone et al. (1978); Bokhoven et al. (2003); Alimirah et al. (2006); Saranyutanon et al. (2020)
LNCaP-AI	CRPC	Low	Yasumizu et al. (2020)
PC3	CRPC	Low	Kaighn et al. (1979); Bokhoven et al. (2003); Alimirah et al. (2006); Saranyutanon et al. (2020)
VCaP	CRPC	High	Korenchuk et al. (2001); Bokhoven et al. (2003); Saranyutanon et al. (2020)
22Rv1	CRPC	High	Sramkoski et al. (1999); Bokhoven et al. (2003); Saranyutanon et al. (2020)
NCI-H660	Neuroendocrine PCa	Low	Carney et al. (1985); Johnson et al. (1989); Bokhoven et al. (2003)

The frequency and grouping of genomic aberrations vary depending on the stage of the disease (He et al., 2022). Hormone sensitive stages are characterized by *SPOP*, *MED12,* and *FOXA1* mutations, the *TMPRSS2::ERG* gene fusion, and *PTEN* deletions or mutations (Abeshouse et al., 2015; He et al., 2022). As the disease advances, the frequency of *TP53* deletions or mutations and *MYC* amplification or overexpression increases (Abeshouse et al., 2015; Quigley et al., 2018; He et al., 2022). CRPC presents with *AR* amplification, mutation, overexpression and/or enhanced signaling, and *APC* deletions or mutations (Abeshouse et al., 2015; Fraser et al., 2017; He et al., 2022). Finally, as the disease advances into the metastatic stage, *RB1* deletions and mutations, *CTNNB1* amplification or overexpression, and *CDK12*, *BRCA1*, *BRCA2*, and *ATM* deletions or mutations become more frequent (Abeshouse et al., 2015; Fraser et al., 2017; Stopsack et al., 2020; He et al., 2022). Overall, regardless of the stage of the disease, mutations or deletions of well stablished tumor suppressors (*PTEN*, *SPOP*, *TP53*, *BRCA1/2*, and *RB1*) or genes that encode for proteins involved in DNA damage repair (*ATM*) and cell cycle control (*MED12*, *CDK12*, and *APC*) are common; while genes that are transcriptional regulators (*FOXA1*, *TMPRSS2::ERG* fusion, *MYC*, *AR*, and *CTNNB1*) present with gain-of-function, amplification or overexpression.

Epigenetic regulators are currently in the spotlight as alternative therapeutic targets in PCa; however, the epigenetic landscape is not fully defined for the various PCa stages. Most epigenetic characterization has focused on histone methylation/acetylation, and DNA hypermethylation (Stelloo et al., 2018; Sugiura et al., 2021; He et al., 2022). The repressive histone marks H3K9me2 and H3K9me3 are reduced in malignant tissues compared to healthy tissues in part as result of overexpression of LSD1, a histone demethylase, which cooperates with AR to promote the expression of cell cycle-associated genes (Metzger et al., 2005; He et al., 2022). In contrast, histone deacetylases, which silence gene expression, are also overexpressed in PCa and their expression is correlated with higher Gleason scores (Weichert et al., 2008). Similarly, the H3K27me3 repressive marks are increased in PCa, which is driven by EZH2 overexpression (Park et al., 2021; He et al., 2022). In terms of DNA hypermethylation, 22% of PCa tumors have been shown to be associated with this feature and DNA methyl transferase inhibitors such as azacytidine and decitabine have been in clinical trials for PCa (He et al., 2022; Zhao et al., 2020). Hypermethylation may result in part from a loss in CTCF (CCCTC-binding factor) in a subset of PCa cases (Damaschke et al., 2020). CTCF is a chromatin insulator and its high expression is, paradoxically, also correlated with a worse prognosis in PCa (Höflmayer et al., 2020) and has been implicated in binding to single nucleotide polymorphisms associated with increased PCa risk (Tian et al., 2023; Guo et al., 2018). CTCF cooperates with cohesin to establish a 3D chromatin architecture consisting of chromatin loops and topologically associating domains, which is important for gene insulation as well as enhancer-promoter interactions that are increased for many genes during PCa progression (Ramanand et al., 2020). For instance, AR binds primarily at enhancers and induces chromatin looping with the *KLK3* promoter to facilitate RNA polymerase II binding (Wanget al., 2005). Many of these loops, which increase in number during PCa progression, require CTCF binding to domain boundaries within the *KLK* family locus (Khoury et al., 2020). Additional transcriptional drivers of PCa similarly rely on enhancer-driven transcription, implicating similar changes in genome organization during PCa progression, which may similarly be dependent on CTCF. Metastatic PCa is also characterized by a general increase in chromatin accessibility (Stelloo et al., 2015), which may facilitate rapid changes in genome organization during progression and provide transcriptional plasticity for the development of therapy resistance.

Chromatin accessibility is facilitated by chromatin remodelers, which mobilize nucleosomes on DNA to repress or activate target genes (Clapier et al., 2017). The SWI/SNF multi-subunit complexes (also called BRG/BRM-associated (BAF) complexes) are ATP-dependent chromatin remodelers important in development and disease (Clapier et al., 2017). Three biochemically distinct SWI/SNF complexes have been defined up to date: canonical BAF (cBAF), polybromo-associated BAF (PBAF), and the non-canonical GLTSCR1/1L-BAF (GBAF or ncBAF) (Alpsoy and Dykhuizen, 2018; Gatchalian et al., 2018; Mashtalir et al., 2018; Wang et al., 2019) (Figure 2). Variations in SWI/SNF subcomplexes that result from cell type-specific expression of paralogs have also been defined: for example, embryonic stem cell-specific BAF (esBAF) (Ho et al., 2009), neural progenitor BAF (npBAF) and neuronal BAF (nBAF) (Wu et al., 2007; Cenik and Shilatifard, 2020; Son and Crabtree, 2014) (Figure 2). All BAF subcomplexes contain a catalytic motor module comprised of the ATPase SMARCA2/4 (SWI/SNF related, Matrix associated, Actin dependent Regulator of Chromatin, subfamily A, member 2/4) connected via its HSA (Helicase-SANT–Associated) domain to ACTL6A or B (actin-like 6A/B), ACTB (β-actin), and BCL7A/B/C (B-cell CLL/lymphoma 7 protein family member A/B/C). This motor module is connected to a core comprised of a dimer of SMARCC1/2 subunits, and SMARCD1/2/3 (Mashtalir et al., 2018; Alpsoy and Dykhuizen, 2018; Gatchalian et al., 2018; Wang et al., 2019; Cenik and Shilatifard, 2020; Son and Crabtree, 2014; He et al., 2021; Dietrich et al., 2023). The incorporation of ARID1A/B, ARID2, or GLTSCR1/1L on to the core seeds the assembly of additional subunits to form cBAF, PBAF, or GBAF (Figure 2). Subunits unique to a particular complex are involved in modulating enzymatic remodeling activity, recognizing DNA/histone substrates, recruiting additional transcriptional regulators or promoting heterotypic phase separation (Kuang et al., 2021; Davis et al., 2023; Patil et al., 2023). The HUGO Gene names and the many aliases of SWI/SNF subunits are listed in Table 2.

**FIGURE 2:**
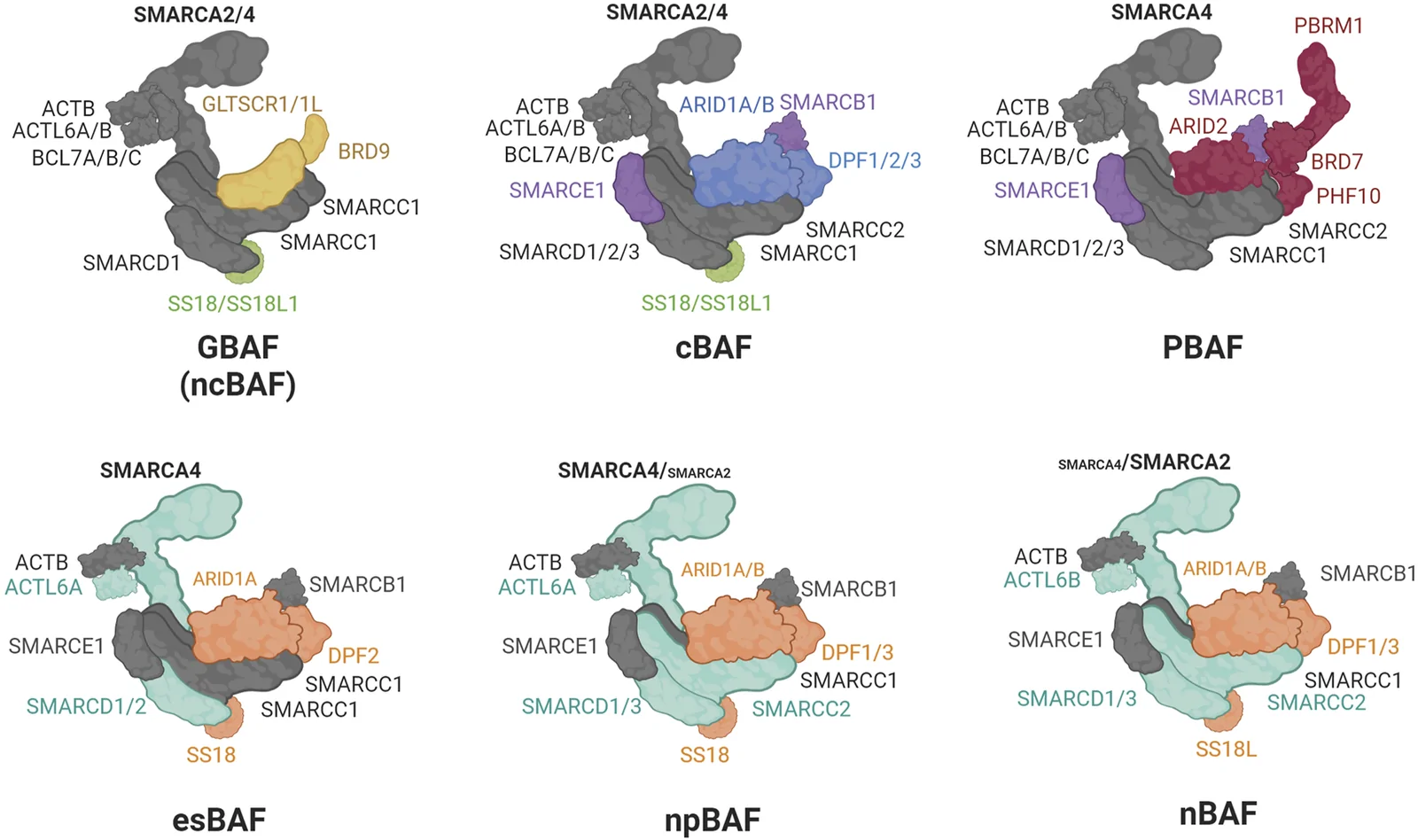
Composition of the SWI/SNF complexes. In gray: shared core subunits, in light green: subunits shared by GBAF and cBAF; in purple: subunits shared by cBAF and PBAF; in yellow: GBAF unique subunits; in blue: cBAF unique subunits; in red: PBAF unique subunits; in cyan: motor and core subunits whose paralogs interchange between esBAF, npBAF, and nBAF; in orange: accessory subunits whose paralogs interchange between esBAF, npBAF, and nBAF.

**TABLE 2:** HUGO names of the SWI/SNF subunits in alphabetical order, the complex(es) they are part of, and their aliases.

HUGO name	Associated complex	Other aliases
ACTB	All	β-actin
ACTL6A	All	BAF53A, SMARCN1
ACTL6B	All	BAF53B, SMARCN2
ARID1A	cBAF	BAF250a
ARID1B	cBAF	BAF250b
ARID2	PBAF	BAF200, Zipzap/p200
BCL7A	All	SMARCJ1
BCL7B	All	SMARCJ2
BCL7C	All	SMARCJ3
BICRA	GBAF	GLTSCR1
BICRAL	GBAF	GLTSCR1L
BRD7	PBAF	-
BRD9	GBAF	-
DPF1	cBAF	BAF45B
DPF2	cBAF	BAF45D
DPF3	cBAF	BAF45C
PBRM1	PBAF	BAF180
PHF10	PBAF	BAF45A
SMARCA2	cBAF and GBAF	BRM
SMARCA4	All	BRG1
SMARCB1	cBAF and PBAF	BAF47, INI-1, SNF5
SMARCC1	All	BAF155, SRG3
SMARCC2	cBAF and PBAF	BAF170
SMARCD1	All	BAF60A
SMARCD2	cBAF and PBAF	BAF60B
SMARCD3	cBAF and PBAF	BAF60C
SMARCE1	cBAF and PBAF	BAF57
SS18	cBAF and GBAF	SYT, SMARCL1
SS18L1	cBAF and GBAF	CREST, SMARCL2

Subunits of the SWI/SNF complex are mutated in around 20% of cancers and several are recognized as tumor suppressors (Kadoch et al., 2013; Shain and Pollack, 2013); however, SWI/SNF mutations are infrequent in PCa. Instead, as described below, aberrant expression of multiple subunits is found in PCa and is often associated with a specific stage. In this review, we summarize the often-conflicting roles of SWI/SNF complexes in PCa, where both tumor suppressive and promoting roles have been reported for several subunits. We focus on how the SWI/SNF complexes facilitate the activity of different PCa key drivers and dependencies such as AR and FOXA1, their stage-dependent functions as the disease advances, and the role they play in the increasingly essential tumor microenvironment (TME).

## 2 SWI/SNF complexes and androgen receptor (AR)-mediated transcription

The central regulator in PCa is AR, a ligand-dependent nuclear transcription factor that contains an N-terminal domain (NTD), a DNA-binding domain (DBD), a C-terminal ligand-binding domain (LBD), and a hinge region that connects the DBD and the LBD (reviewed in Dai et al., 2017). When unbound, AR is found in the cytoplasm with chaperone proteins (i.e., HSP90) that prevent its degradation. When AR binds to an androgen ligand, mainly dihydrotestosterone (DHT), it undergoes a conformational change leading to its dissociation from the chaperones, homodimerization, and translocation to the nucleus. There it associates with transcriptional coregulators that facilitate its binding to androgen response elements (AREs) in enhancers and promote looping with promoters to allow for transcription of genes important for cell survival and cell proliferation (Figure 3) (Royen et al., 2012; Shafi et al., 2013; Dai et al., 2023). AR is not typically altered during the initiation of PCa; however, the dependency of PCa on AR-mediated transcription has made it the major focus of therapies.

**FIGURE 3:**
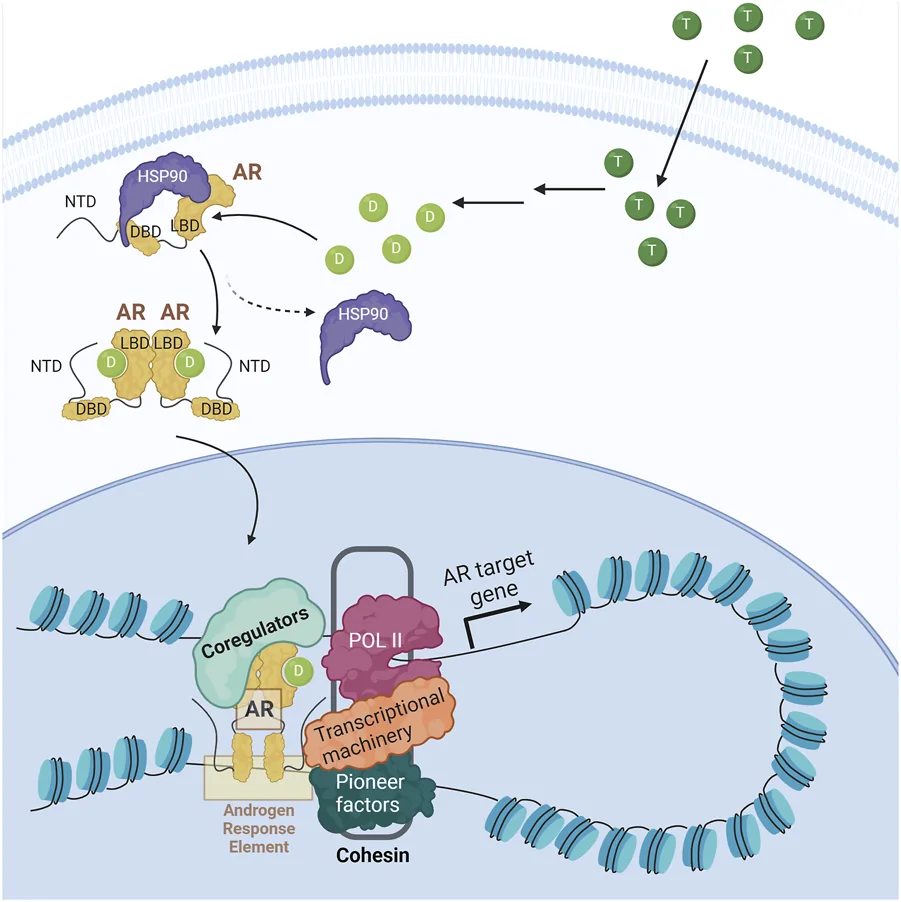
Androgen receptor signaling. Upon entering the cell, testosterone (dark green circle) is metabolized into dihydrotestosterone (light green circle), the main AR ligand. Upon ligand binding, AR is released from the chaperone (i.e., HSP90), dimerizes and translocates to the nucleus where, along with coregulators and RNA polymerase II (POL II), promotes AR target genes expression.

### 2.1 Early studies investigating AR dependence on SWI/SNF

Initial studies investigating SWI/SNF involvement in AR-mediated transcription utilized ectopic expression of AR and exogenous activity reporters such as probasin pARR2-luciferase, PSA-luciferase, and the mouse mammary tumor virus promoter-driven luciferase (MMTV) (Inoue et al., 2002; Marshall et al., 2003; Link et al., 2005; Klokk et al., 2007; Kikuchi et al., 2009; Jin et al., 2018). In these systems, the ATPase subunit SMARCA2 is recruited by AR for chromatin remodeling and AR reporter activity (Y. Dai et al., 2008; Klokk et al., 2007; Marshall et al., 2003). In Smarca2^−/−^ mice, however, AR activity is not affected (Shen et al., 2008), raising a question of SMARCA2 dependence in PCa. In a small patient cohort, SMARCA2 levels were increased in PCa samples compared to normal tissue (Sun et al., 2007); however, according to large scale genomics and proteomic studies, contrary to what had been originally reported, SMARCA2 levels are decreased in PCa patients compared to normal prostate tissue (Shen et al., 2008; Muthuswami et al., 2019) and SMARCA2 expression is inversely correlated with the advancement of the disease.

Other reporter studies identified a dependency on SMARCA4 for AR activity (Huang et al., 2003), which were further characterized to interact using co-immunoprecipitation (Li et al., 2006), and other techniques (Lempiäinen et al., 2017; Paltoglou et al., 2017; Stelloo et al., 2018; Launonen et al., 2021). SMARCA4 associates with AR when the NTD is associated with the LBD (Li et al., 2006), which is a stronger interaction if the polyglutamine (polyQ) tract located in the NTD is shorter (Q. Wang et al., 2004) (Figure 4). The polyQ tract is encoded by a series of CAG triplets located in exon 1 of *AR* which may vary in length between individuals (He et al., 2020). AR with shorter polyQ tract is associated with an increase in AR transcriptional activity and higher risk and aggressiveness in PCa (He et al., 2020), implicating SMARCA4 in promoting AR activity and PCa advancement. Some early studies found SMARCA4 upregulated in PCa samples (Sun et al., 2007) and unlike SMARCA2, this upregulation has been confirmed in larger datasets (Muthuswami et al., 2019).

**FIGURE 4:**
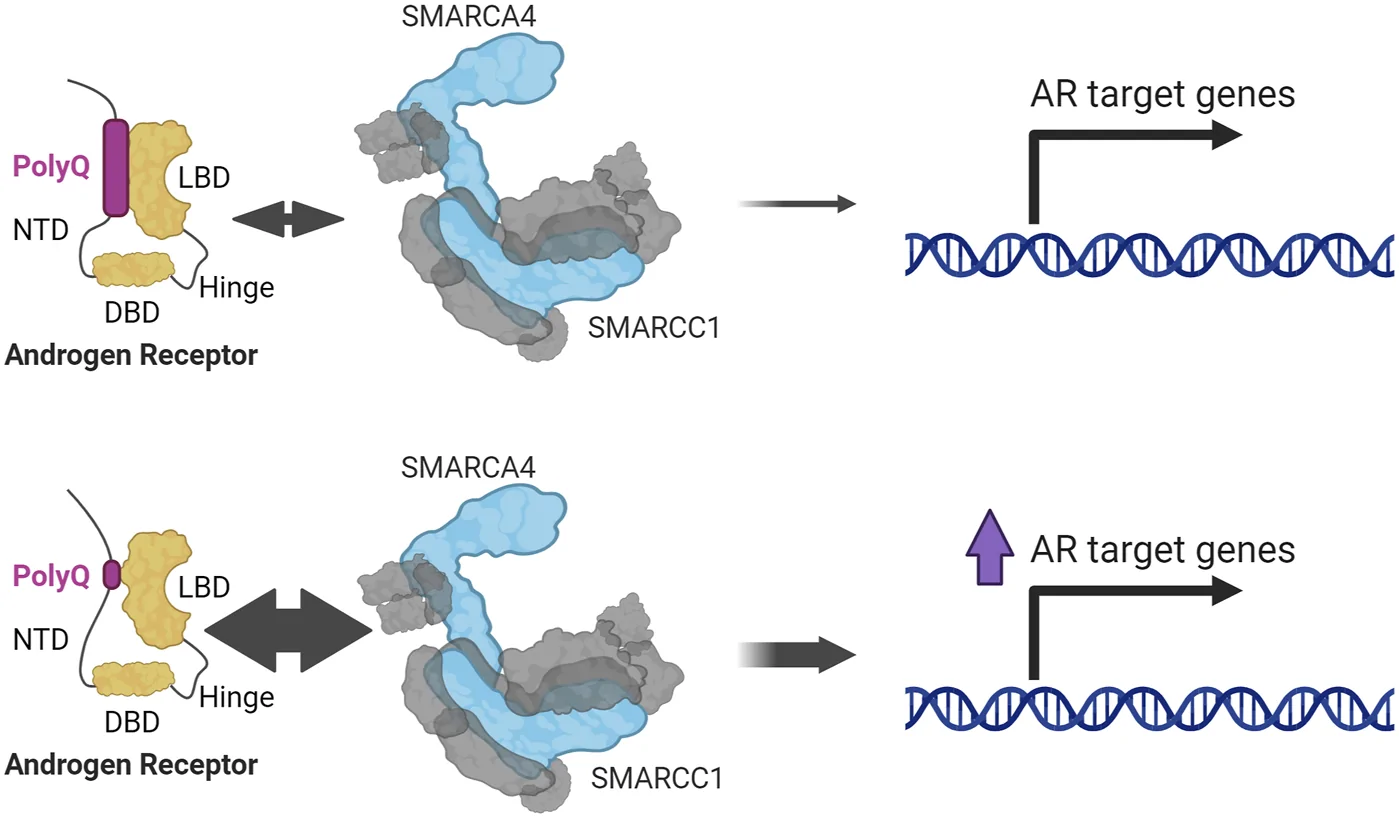
SMARCA4 and SMARCC1 interact with AR through the interaction between its NTD (N-terminal domain) and LBD (ligand binding domain). This interaction is stronger the shorter the polyglutamine tract (PolyQ) (Li et al., 2006; Wang et al., 2004).

The core subunit SMARCC1 was one of the first subunits other than the ATPase subunits established to be important for AR signaling (Wang et al., 2004; Link et al., 2005; Hong et al., 2005) (Figure 4). This function is typically dependent on the ATPase; however, one study concludes that Smarcc1 can promote an AR luciferase reporter through an ATPase subunit-independent association between Smarcc1 and AR (Hong et al., 2005). Proteomic analyses consistently identified the ATPases, SMARCC1, and other SWI/SNF subunits with AR, (Lempiäinen et al., 2017; Paltoglou et al., 2017; Stelloo et al., 2018; Launonen et al., 2021; Ban et al., 2021); however, the absence of the ATPase subunits in this study might indicate that AR association with SWI/SNF is not necessarily mediated through the ATPase module.

The last subunit identified in these early studies is SMARCE1, a subunit shared by cBAF and PBAF. It is required for endogenous AR activity and immunoprecipitates with endogenous AR independent of ligand binding (Link et al., 2005). Later studies showed that SMARCE1 is temporarily recruited to AREs and, in biochemical assays, binds to the DBD and hinge region of AR (Link et al., 2008). Inhibition of the interaction between SMARCE1 and AR using an AR N-terminal peptide abrogates AR activity and reduces AR-dependent PCa proliferation, indicating SMARCE1 promotes AR activity (Link et al., 2005). After these early studies, the role for several other subunits in AR signaling has been reported. In the following sections, we describe the different aspects of AR signaling that require SWI/SNF.

### 2.2 SWI/SNF subunits interact with AR

Via distinct methodologies, proteomic studies in PCa cells have confirmed that AR interacts with multiple SWI/SNF subunits (Lempiäinen et al., 2017; Paltoglou et al., 2017; Stelloo et al., 2018; Launonen et al., 2021; Ban et al., 2021) (Table 3). SMARCA4 was identified in all studies while SMARCA2 was not, perhaps due to its reduced expression in PCa. As for the shared core subunits, SMARCC1/2, SMARCD1/2/3, and ACTL6A were identified, as were SMARCB1 and SMARCE1, shared by cBAF and PBAF. cBAF-specific subunits ARID1A/B and PBAF-specific subunits PBRM1 and ARID2 were identified; however, GBAF subunits SS18/SS18L1, GLTSCR1/1L, and BRD9, were not. These data indicate that PBAF and cBAF function as direct AR coactivators, while the role of GBAF in PCa is independent from a direct interaction with AR.

**TABLE 3:** Summary of proteomic studies evaluating binding to SWI/SNF subunits to AR. Blue shade indicates the subunit was present in the proteomic analysis (Lempiäinen et al., 2017; Paltoglou et al., 2017; Stelloo et al., 2018; Launonen et al., 2021; Ban et al., 2021).

	Lempiäinen (2017)	Paltoglou (2017)	Stelloo et al. (2018)	Launonen et al. (2021)	Ban et al. (2021)
Cells	HEK293T - ectopic AR	R1-AD1	LNCaP	VCaP	LNCaP
Technique	proximity-dependent biotin identification	RIME	RIME	ChIP-SICAP	RIME
Subunit					
*Shared core subunits*					
SMARCA2					
SMARCA4					
SMARCC1					
SMARCC2					
SMARCD1					
SMARCD2					
SMARCD3					
ACTL6A					
*cBAF unique subunits*					
ARID1A					
ARID1B					
*PBAF and cBAF shared subunits*					
SMARCB1					
SMARCE1					
*PBAF unique subunits*					
PBRM1					
ARID2					

Considering the strong data supporting SWI/SNF association with AR, understanding the biochemical basis for this association is important for deciphering the role of SWI/SNF in CRPC. Most AR inhibitors target the c-terminal LBD; however some forms of CRPC express constitutively active AR variants without the LBD (Li et al., 2013). Alternate strategies for these cancers include the development of drugs targeting the NTD (Ji et al., 2023; Dai etbal., 2023). One NTD inhibitor is VPC-220010, which inhibits the association between AR and several important co-regulators to reduce proliferation of LNCaP and 22Rv1 cells (Ban et al., 2021). VPC-220010 inhibits the androgen-dependent interaction between AR and SMARCC1, SMARCC2, SMARCD1, SMARCD2, and SMARCA2, but not SMARCA4 (Ban et al., 2021). This indicates that SWI/SNF associates with AR through the NTD, but the interaction between SMARCA4 and AR may occur through the LBD, as previously demonstrated (Li et al., 2006). While SWI/SNF association is likely stronger for wild type AR, it may also contribute to the activity of AR splice variants in CRPC.

### 2.3 AR target gene expression regulation by SWI/SNF

In the vast majority of PCas the genes *KLK3* (PSA), *KLK2*, *TMPRSS2*, *FKBP5*, *NKX3.1*, *LSD1*, and *UBE2C* are upregulated by AR (Takayama and Inoue, 2013). A SMARCA2/4 dual degrader reduces AR target gene expression in multiple PCa cell lines (Xiao et al., 2022). Core subunits shared by all three SWI/SNF complexes (ACTL6A, SMARCD1, and SMARCC1) or cBAF and PBAF complexes (SMARCE1 and SMARCD2) are similarly required for AR target gene expression (S. Liu et al., 2017; Wijngaart et al., 2009; Sun et al., 2011; Ertl et al., 2023). cBAF complexes, which are the most abundant, are thought to be the primary dependency (Xiao et al., 2022). Several studies implicate SMARCA4 as the primary ATPase for AR gene regulation. SMARCA4 and SMARCC1 regulate the second largest number of androgen-dependent AR target genes after p300, with a significantly larger number than SMARCA2 (S. Liu et al., 2017) (Figure 5). Confirming a role for SMARCA4 as the major AR co-regulator, ACTL6A is recruited to two AREs of *KLK3* with SMARCA4, but not SMARCA2 (Jin, Kim, and Jeong, 2018) (Figure 5) and SMARCA4, but not SMARCA2, was enriched at the second intron of the AR target gene *ITGA2* (Balasubramaniam et al., 2013). At the *FKBP5* locus in VCaP cells, three validated AREs have high SMARCA2, but not SMARCA4, enrichment (Ban et al., 2021), indicating that SMARCA2 may be required for at least a subset of AR target genes (Lempiäinen et al., 2017; Ban et al., 2021; Launonen et al., 2021) (Figure 5). Supporting a role for both ATPases in PCa, loss of both SMARCA4 and SMARCA2 is required for decreased chromatin accessibility at enhancers, including those occupied by AR (Xiao et al., 2022).

**FIGURE 5:**
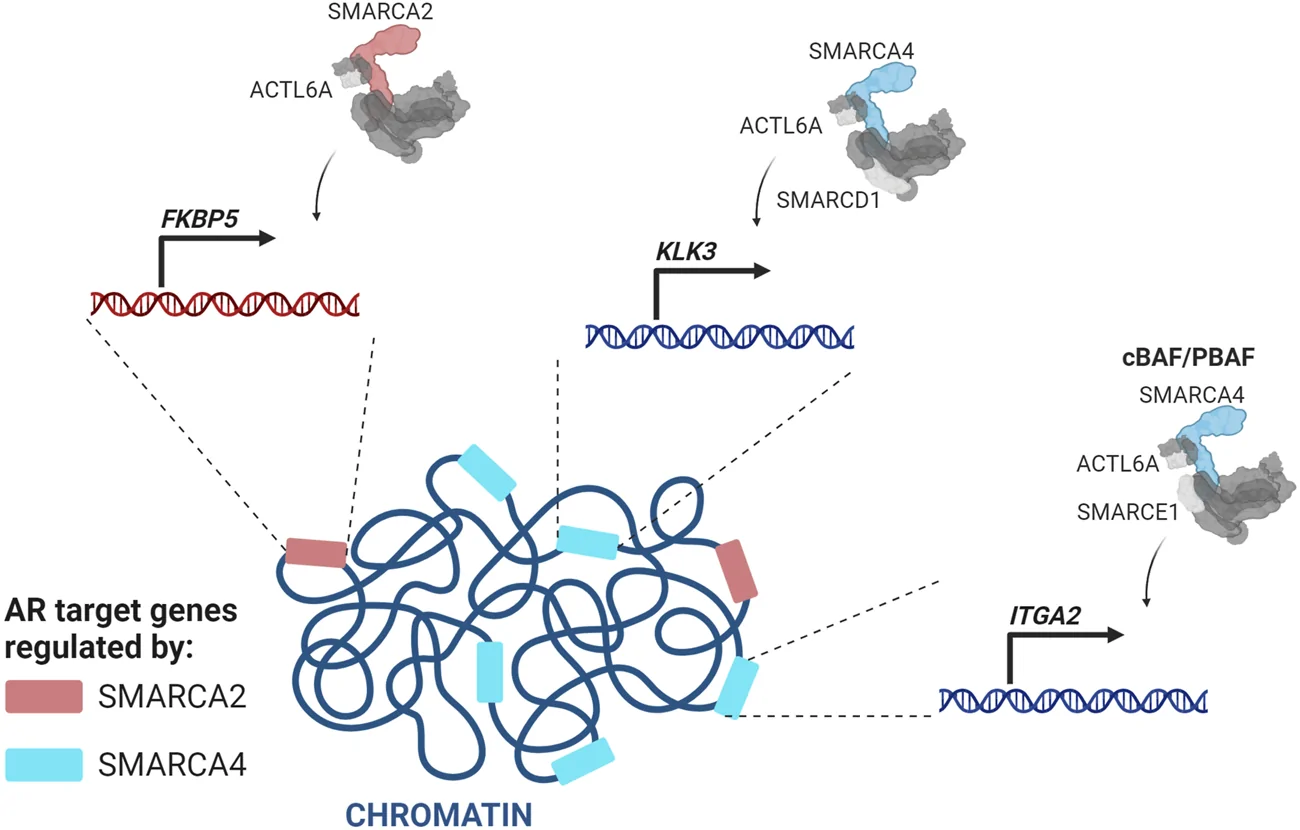
SMARCA4-and SMARCA2-containing SWI/SNF complexes regulate different sets of AR target genes (Makkonen et al., 2009; Balasubramaniam et al., 2013; Jin, Kim, and Jeong, 2018; Wijngaart et al., 2009; S. Liu et al., 2017).

### 2.4 Bromodomain (BD)-containing proteins and AR regulation

In PCa the most studied bromodomain-containing proteins are the BET (bromodomain and extra-terminal domain) proteins, primarily BRD4 (Urbanucci and Mills, 2018); however, the BDs on SWI/SNF subunits such as BRD9, SMARCA4, SMARCA2, PBRM1, and BRD7 are also important for AR activity.

BRD9, a unique subunit of the GBAF complex, is required for proliferation of AR-positive cell lines, and high GBAF subunit expression correlates with a decrease in tumor-free survival in patients (Alpsoy et al., 2021). BRD9 regulates the expression of AR target genes in both androgen-responsive and CRPC cell lines (Alpsoy et al., 2021) through association with BRD4. BRD9 colocalizes with BRD4 and CTCF at multiple genomic sites including at AR target genes (Alpsoy et al., 2021). This is presumably to facilitate chromatin looping and enhancer activation; however, further studies are needed to define the function of GBAF in PCa.

A role in CTCF-mediated looping in PCa may extend to other SWI/SNF subcomplexes as subunits from all three SWI/SNF complexes immunoprecipitate with CTCF (Valletta et al., 2020). Using publicly available datasets for Hi-C chromosomal contacts, histone modifications, DNA methylation, and nucleosome positioning in LNCaP cells (Giles et al., 2019), SMARCA4 was associated more with “actively marked” chromatin and CTCF enrichment than SMARCA2, which was more associated with “repressively marked” chromatin (Giles et al., 2019). This is in accordance with the model that SMARCA4-containing SWI/SNF complexes target different sites than SMARCA2-containing complexes. Further studies focused on the loci where unique subunits colocalize with CTCF upon AR activation in PCa cells could shed light on the mechanism by which SMARCA2 and SMARCA4 regulate different sets of AR target genes.

Another BD-containing protein explored in PCa is TRIM24, which promotes AR signaling and has increased expression in CRPC (Groner et al., 2016). BRD7, a PBAF subunit, interacts with TRIM24 to limit TRIM24-induced AR transcriptional activity in a luciferase reporter assay (Kikuchi et al., 2009); however, this interaction was not studied at an endogenous level. In our recent work, BRD7 knockdown reduces proliferation of AR-dependent PCa cell lines and BRD7 BD inhibition in LNCaP cells downregulates the expression of AR target genes (Ordonez-Rubiano et al., 2023). Similarly, in the near-haploid HAP1 cells loss of BRD7 or ARID2 leads to reduction in genes involved in response to testosterone and dehydroepiandrosterone (Schick et al., 2019). Further supporting a role for PBAF in PCa, PBRM1 is almost 10 times more highly expressed in PCa than in benign prostatic hyperplasia (Mota et al., 2019). LNCaP cells (AR-dependent) have higher PBRM1 levels compared to PC-3 (AR-independent), DU145 (AR-independent), and RWPE-1 (normal prostate) cells (Mota et al., 2019). Supporting a role for PBAF in AR-mediated function, a PBRM1-selective BD inhibitor reduced the growth of LNCaP cells but not PC-3 and RWPE-1 cells (Shishodia et al., 2022). Overall, increasing evidence supports PBAF in the positive regulation of AR signaling; however, the mechanism by which this occurs is unexplored.

### 2.5 Regulation of AR expression and protein stability by SWI/SNF

Most studies focus on the regulation of AR-mediated transcription, but fewer studies investigate the regulation of AR itself. Consequently, little is known about the function of SWI/SNF in AR expression regulation except that long term loss of SWI/SNF results in a decrease in AR, which could be direct or indirect (Xiao et al., 2022). SMARCA4 is associated with Protein arginine methyltransferase 5 (PRMT5) and SP1 at the *AR* promoter where PRMT5 is required for transcription (Deng et al., 2017); however, whether AR transcription is dependent on chromatin remodeling by SMARCA4 has not been determined (Figure 6).

**FIGURE 6:**
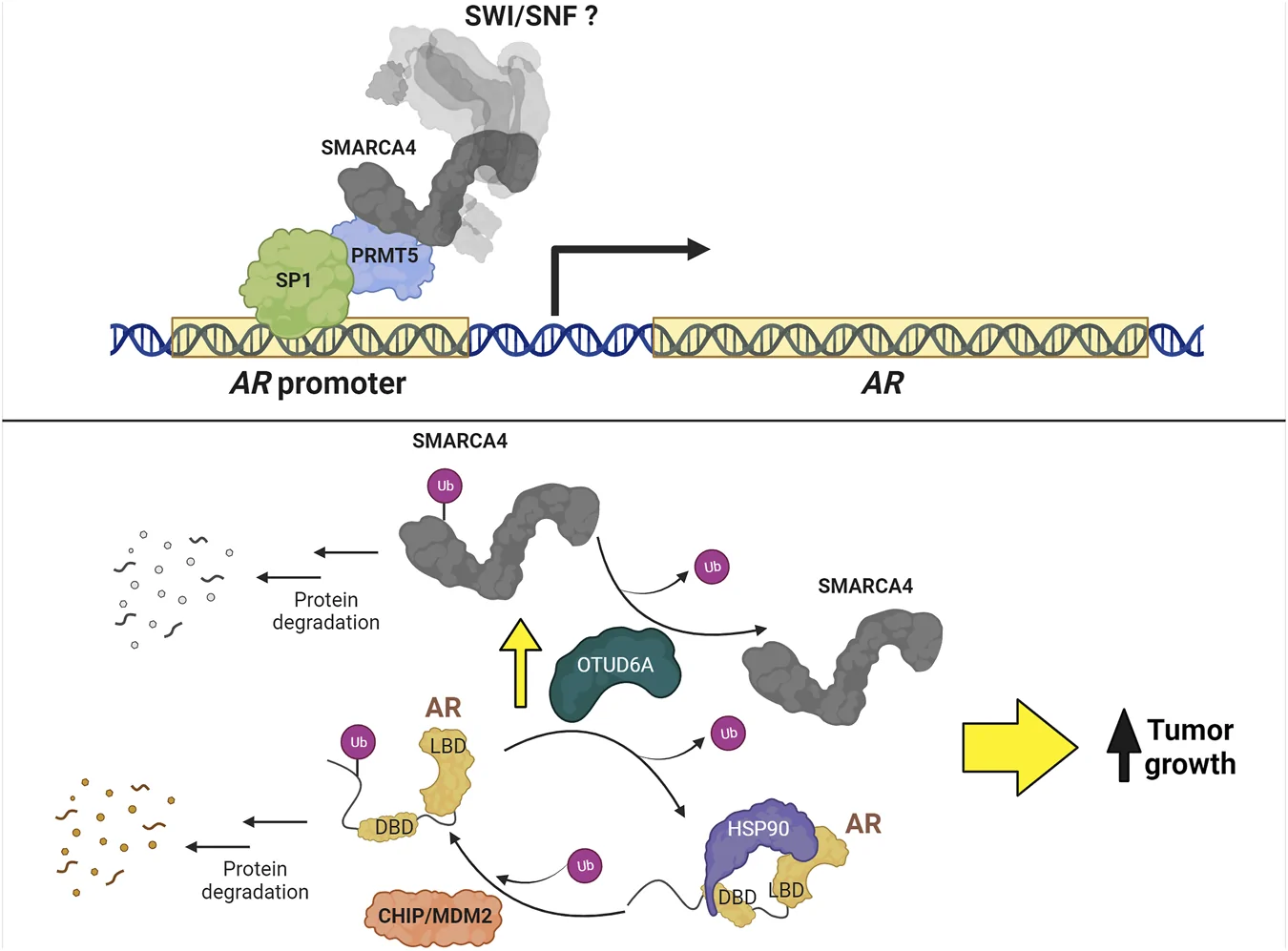
Regulation of AR expression in PCa. Top: PRMT5 forms a complex with SP1 and SMARCA4 to promote *AR* transcription. Bottom: MDM2 and CHIP ubiquitinate AR to promote protein degradation. Conversely, the protein levels of AR and SMARCA4 are upregulated by OTUD6A-mediated deubiquitination, thereby increasing tumor growth.

The regulation of AR stability has been a subject of investigation for many years. For example, CHIP (C-terminus of Hsp70-interacting protein) (Sarkar et al., 2014) and MDM2 (mouse double minute 2 homolog) (Gaughan et al., 2005; Giridhar et al., 2019) ubiquitin ligases promote AR degradation via proteasomal activity, while the long non-coding RNA PCBP1-AS1 prevents AR degradation by stabilizing a complex formed between AR and the deubiquitinase USP22 (Zhang et al., 2021); however, no connection has been made between these ubiquitin-mediated processes and SWI/SNF. In contrast, OTUD6A (ovarian tumor deubiquitinase 6A), a deubiquitinase amplified in PCa patients that promotes tumor growth in mice, deubiquitinates both SMARCA4 at K27 and AR at K11 to prevent their degradation by the proteasome (Fu et al., 2022) (Figure 6). Although promising as a strategy to promote degradation of SMARCA4 and AR simultaneously in PCa cells, no OTUD6A inhibitor has been developed.

### 2.6 Other AR coregulators and their relationship with SWI/SNF

AR transcriptional coregulators are mainly involved in four different roles: chromatin/histone modification, co/chaperone, scaffolding, and transcription machinery assembly (Leach, Fernandes, and Bevan, 2022). These components can be classified as coactivators or corepressors depending on their function in regulating AR transcriptional activity (Heemers and Tindall, 2007; Leach, Fernandes, and Bevan, 2022). There is increasing evidence supporting target gene-specific coregulator activity, wherein next generation sequencing techniques reveal that individual AR coregulators can have positive, negative, or no effects on specific AR target genes (Leach et al., 2022; Liu et al., 2017). This observation aligns with the SWI/SNF roles discussed in previous sections, in particular, that SMARCA4- and SMARCA2-containing complexes regulate separate sets of AR target genes. In this section we discuss the relationship between SWI/SNF and three less defined AR coregulators: ZMIZ1, ZMIZ2, and HMGB1.

ZMIZ1 and ZMIZ2 (Zinc Finger MIZ-Type Containing 1/2) are members of the protein inhibitor of activated STAT (PIAS) family of proteins that promote the activity of multiple signaling pathways, including AR (Lomelí, 2022). Immunoprecipitation studies indicate that SMARCA4 and SMARCE1 interact with ZMIZ2 and cooperate to promote AR transcriptional activity (Huang et al., 2005). The ability of ZMIZ1 to promote the activity of AR with short polyQ tract is enhanced by SMARCA4 and SMARCE1 (X. Li et al., 2011), further supporting the role of SWI/SNF in promoting transcriptional activity of AR with short polyQ tract.

High-mobility group box 1 (HMGB1) is an architectural chromatin-binding factor that facilitates transcription, recombination, and DNA damage repair (Xue et al., 2021) and *HMGB1* single nucleotide polymorphisms may predict PCa progression (Chou et al., 2020). HMGB1 can also be released from cells and its co-expression with the inflammatory receptor Receptor for Advanced Glycation End products (RAGE) correlates with progression and worse overall survival in PCa patients (Zhao et al., 2014). SMARCA4 co-immunoprecipitates with HMGB1 in both PC-3 and LNCaP cells, and colocalizes with HMGB1 in the nucleus (Lv et al., 2019). HMGB1 regulates SMARCA4 expression, and by inducing SMARCA4 expression in the AR-negative PC-3 cells, HMGB1 promotes epithelial-mesenchymal transition (EMT) (Lv et al., 2019). HMGB1 also directly interacts with AR and can reactivate AR signaling (Chen et al., 2022) indicating that SMARCA4 cooperates with HMGB1 to facilitate AR signaling in early stages of PCa, while in advanced CRPC, SMARCA4 cooperates with HMGB1 to promote a different set of genes involved in EMT. Comparing whole genome sequencing data between models of hormone sensitive and castration resistant disease could shed light on how this reprogramming may be occurring.

## 3 The interplay between SWI/SNF complexes and genetic drivers of PCa

While there is no universal genetic driver of PCa, several factors are mutated or altered in a significant portion of patients to drive the initiation and progression of PCa development and represent dependencies in PCa. Hereby we summarize discoveries on how SWI/SNF is involved in these additional events.

### 3.1 TMPRSS2::ERG fusion

ETS (E-26 transformation-specific)-related gene (ERG) is an oncogenic transcription factor that is overexpressed in multiple cancers such as Ewing’s sarcoma, hematologic malignancies, and PCa (Adamo and Ladomery, 2016). Multiple gene fusions are present in PCa, the most frequent being TMPRSS2::ERG (Lorenzin and Demichelis, 2022). The TMPRSS2::ERG fusion gene in PCa was first reported in 2005 as a recurrent chromosomal rearrangement in patients (Tomlins et al., 2005) which increases ERG expression to induce ERG target gene expression (Fujita and Nonomura, 2018; Z. Wang et al., 2017; Tomlins et al., 2005). Since then, it has been established as a urinary biomarker of poor prognosis of PCa patients (Laxman et al., 2006; Sanda et al., 2017). IP-MS identified cBAF subunits as ERG interactors (Fu et al., 2021; Sandoval and Pulice, 2018). ChIP-Seq studies indicate that ERG dictates the overall targeting of cBAF, and that, conversely, the binding of ERG to chromatin depends on cBAF activity (Sandoval and Pulice, 2018). Moreover, SMARCA4 knockdown in organoids derived from prostate epithelia from *Pten* ^+/−^, *Tmprss2::ERG* ^+^ mice prevents basal-to-luminal transition, suggesting the interplay between SWI/SNF and TMPRSS2::ERG could drive PCa oncogenesis. The TMPRSS2::ERG fusion gene is thought to promote cell cycle progression, as knocking down *ERG* leads to G_0_/G_1_ cell cycle arrest in VCaP cells (Wang et al., 2017). Whether the TMPRSS2::ERG—SWI/SNF interaction drives such function in cell cycle progression of PCa cells remains to be elucidated.

### 3.2 FOXA1

Forkhead box A1 (FOXA1) is a pioneer factor that is recruited to chromatin in the presence of the H3K4me1 and H3K4me2 histone marks and induces chromatin accessibility at loci with reduced DNA methylation (Lupien et al., 2008; Sérandour et al., 2011). FOXA1 is highly mutated in PCa and drives PCa progression by poising sites for AR binding (Barbieri et al., 2012; Gerhardt et al., 2012) and promoting promoter-enhancer loops at PCa specific genes (Rhie et al., 2019). In addition to AR, FOXA1 is the most enriched transcription factor at SMARCA4 promoter binding sites in LNCaP cells. At the promoters of the AR target genes *KLK2* and *PCAT1*, FOXA1 binding is dependent on SMARCA4 (Giles et al., 2021). Supporting this, PCa cells with high AR and FOXA1 expression are dramatically more sensitive to the SMARCA2/4 degrader AU-15330 compared to cells that lack AR and FOXA1 (Xiao et al., 2022). Treatment of these cells with AU-15330 reduces chromatin accessibility and subsequent binding of both AR and FOXA1 to the enhancers of essential genes (Xiao et al., 2022).

### 3.3 HOXB13

Homeobox protein Hox-B13 (HOXB13) is a transcription factor highly expressed in the prostate involved in its development and maintenance (Kim et al., 2010; Brechka et al., 2017). HOXB13 has demonstrated to be a prognostic marker for PCa with increased expression of AR-variant 7 target genes (Chen et al., 2018; Kim et al., 2020). In LNCaP cells SMARCA4 binding did not enrich for FOXA1 or HOXB13 at AR binding sites (S. Stelloo et al., 2018), while in VCaP cells ATAC-seq analyses indicated FOXA1 and HOXB13 binding motifs were enriched in sites dependent on SMARCA4 for accessibility (Launonen et al., 2021). These results indicate that SMARCA4 may be required for FOXA1 and HOXB13 binding primarily in a CRPC setting.

## 4 SWI/SNF complexes regulate PCa progression

SWI/SNF complexes regulate gene expression in a context-dependent manner, wherein their activity is dependent on the genetic background and disease stage (Kadoch et al., 2013; Cenik and Shilatifard, 2020; Mittal and Roberts, 2020). We described above the role of SWI/SNF in early stages of PCa that are dependent on AR and driven by transcriptional drivers such as TMPRSS2::ERG and FOXA1. In this section we will focus on two additional contexts: progression to the metastatic stage and therapy resistance, including lineage plasticity-associated neuroendocrine differentiation (NED).

### 4.1 SWI/SNF and the emergence of metastatic phenotypes

Other key driver events in PCa include loss of tumor suppressors TP53 and PTEN (Abeshouse et al., 2015; Robinson et al., 2015) and overexpression of EZH2 (Park et al., 2021). CRISPR Cas9-based screening revealed a synthetic lethal relationship between PTEN and SMARCA4 where ablation of SMARCA4 reduced growth of PTEN-null 22Rv1 PCa cells while having no effect in cells expressing wildtype PTEN (Ding et al., 2019). These results were further corroborated in a mouse model where SMARCA4 was demonstrated to be critical for progression of PCa tumors to invasive stages in Pten^PC−/−^ mice (Ding et al., 2019).

In metastatic PCa, SMARCA4 has been connected to the expression of long-chain fatty acid elongase 3 (ELOVL3), which is involved in the synthesis of C20-C24 saturated and mono-unsaturated neutral very long-chain fatty acids (Westerberg et al., 2004). In PCa models, a metastatic phenotype can be induced with androgens (Lin et al., 2018) and TGF-β (Mirzaei et al., 2022). Under these conditions, SMARCA4 promotes migration and invasion of DU145 cells by interacting with p300 and the nuclear receptor RORγ at the *ELOVL3* promoter to induce transcription. Based on a general role for RORγ in cholesterol biosynthesis, SWI/SNF chromatin remodeling may be supporting metastasis and invasion in PCa by regulating the expression of genes involved in lipid metabolism and androgen synthesis (Stuchbery et al., 2017; Zou et al., 2022); however, mechanistic studies would be required to confirm this function genome-wide.

The role of other SWI/SNF subunits in metastatic PCa is less defined, with only a few subunits investigated so far. SMARCE1 is upregulated in prostatic intraepithelial neoplasia compared with benign prostatic hyperplasia tissues and promotes androgen-independent expression of genes involved in cell migration and metastasis (Balasubramaniam et al., 2013). BRD7, a PBAF-specific subunit is overexpressed in the AR-negative cell lines DU145 and PC-3 cells, and the knockdown of BRD7 leads to reduced cell proliferation, migration and invasion (Liang et al., 2019). This could indicate that, in contrast to the cBAF dependency for enhancer-mediated transcription, metastatic gene signatures may be more dependent on PBAF complexes; however, mechanistic studies are required to dissect the roles of distinct SWI/SNF subcomplexes in PCa metastasis.

### 4.2 Treatment-emergent neuroendocrine prostate cancer

Normal prostate architecture consists of luminal cells, basal cells, and interspersed neuroendocrine (NE) cells that surround the lumen (Figure 1). The vast majority of PCa are adenocarcinomas that arise from the luminal cells. Upon prolonged treatment with AR signaling inhibitors, a few of the cancer cells adapt and transdifferentiate to a NE-like state, which is a lineage plasticity event that involves cellular, molecular, and morphological identity changes (Davies et al., 2018; Davies et al., 2023). This transdifferentiation is an important contributor to the development of treatment-emergent neuroendocrine PCa (TE-NEPC) (Beltran et al., 2016), which is a subset of CRPC (10–20%) that is aggressive, AR null, transcriptionally unique, and treatment-resistant, with a median survival of 1 year (Aggarwal et al., 2018; Abida et al., 2019) (Figure 1). Of note, while TE-NEPC is different from focal NEPC (a small cell PCa subtype that arises directly from NE cells of normal prostate) (Small et al., 2016), in the clinic, it is difficult to distinguish between them, as they both present with low PSA levels and the expression of classical NE markers such as CD56, chromogranin A, synaptophysin, and neuron specific enolase (Aggarwal et al., 2014). Tumor tissue matched whole-exome sequencing showed that loss of *RB1* and *TP53* are distinguishing features of TE-NEPC compared to CRPC (Beltran et al., 2016). The somatic landscape of CRPC and TE-NEPC are similar to one another suggesting that epigenetic mechanisms drive the clonal evolution of TE-NEPC from the adenocarcinoma under the selection pressure of anti-androgen therapy (Beltran et al., 2016). Efforts are being made to identify the epigenetic drivers of this process, including SWI/SNF, with an eye on reversing or combating drug resistance (Cheng and Wang, 2021).

The conversion between cell-type specific mammalian SWI/SNF complex subunit configuration is well-established during development. In the differentiation from embryonic stem cells to terminally differentiated neurons, the cBAF subunits (ACTL6A, DPF2, SMARCD1/2, SMARCC1, SMARCA4, and SS18) are exchanged with paralogues (ACTL6B, DPF1/3, SMARCD1/3, SMARCC2, SMARCA2, and SS18L1; respectively) (Olave et al., 2002; Lessard et al., 2007; Ho, Ronan, et al., 2009) (Figure 2). To some extent, this developmental SWI/SNF configuration switch is recapitulated during the transition from CRPC to TE-NEPC (Cyrta et al., 2020), wherein a few neuronal SWI/SNF subunits (DPF1, SS18L1, and ACTL6B) are upregulated both at mRNA and protein levels in a TE-NEPC patient cohort. In contrast to a decrease during neuronal differentiation, SMARCA4 expression is increased in TE-NEPC, and its expression positively correlates to the expression of *SYP* (neuroendocrine marker) and *SOX2* (pluripotency marker) (Cyrta et al., 2020). This suggests that unique SWI/SNF complexes play a role in emergence of TE-NEPC.

Some potential mechanistic understanding of SWI/SNF in NEPC has been revealed through the study of Mucin 1 (MUC1), which is expressed as two separate, non-covalently bound, parts: the N-terminus (MUC1-N) involved in cell-cell adhesion, and the C terminus (MUC1-C) involved in cell signaling and transcription (Kufe, 2023). MUC1 is overexpressed specifically in androgen-independent and neuroendocrine PCa cell lines (LNCaP-AI, DU-145, and NCI-H660) (Yasumizu et al., 2020) and reduced *MUC1-C* expression results in increased expression of AR target genes and decreased expression of NE and pluripotency genes (Yasumizu et al., 2020). MUC1-C binds directly to genomic sites near *SMARCA4*, *ARID1A*, *PBRM1*, *ARID2,* and *BRD7* (Hagiwara et al., 2021; Hagiwara et al., 2021). Reduction in nuclear expression of MUC1-C via genetic knockdown or chemical inhibition using the MUC1-C dimerization inhibitor, GO-203, results in decreased expression of cBAF and PBAF*,* but not GBAF subunits (Hagiwara et al., 2021; Hagiwara et al., 2021). In addition, MUC1-C co-immunoprecipitates with PBRM1 and directly cooperates with PBAF to regulate the expression of NRF2 target genes (Hagiwara et al., 2021). Although these studies provide evidence that SWI/SNF subunits play a role in TE-NEPC, the mechanistic interplay within the sub-complexes remains to be elucidated.

## 5 The crosstalk between SWI/SNF complexes and the tumor microenvironment (TME) in PCa

The prostate TME contains carcinoma-associated fibroblasts (CAFs) and several types of immune cells, including but not limited to, T cells, B cells, NK cells, macrophages, myeloid-derived suppressor cells (MDSCs), and neutrophils (Kwon et al., 2021; Palano et al., 2022) (Figure 7). These cell types all display plasticity and can differentiate into multiple subtypes with different effects on tumor growth and therapy resistance. Because of this, both immune cells and CAFs could be dependent on epigenetic modulation by SWI/SNF for pro or anti-tumorigenic function. Moreover, the effects of SWI/SNF within the cancer cells, including the perturbation of specific subunits, can change the interaction between the PCa and the immune microenvironment (Li et al., 2022; Hagiwara et al., 2022).

**FIGURE 7:**
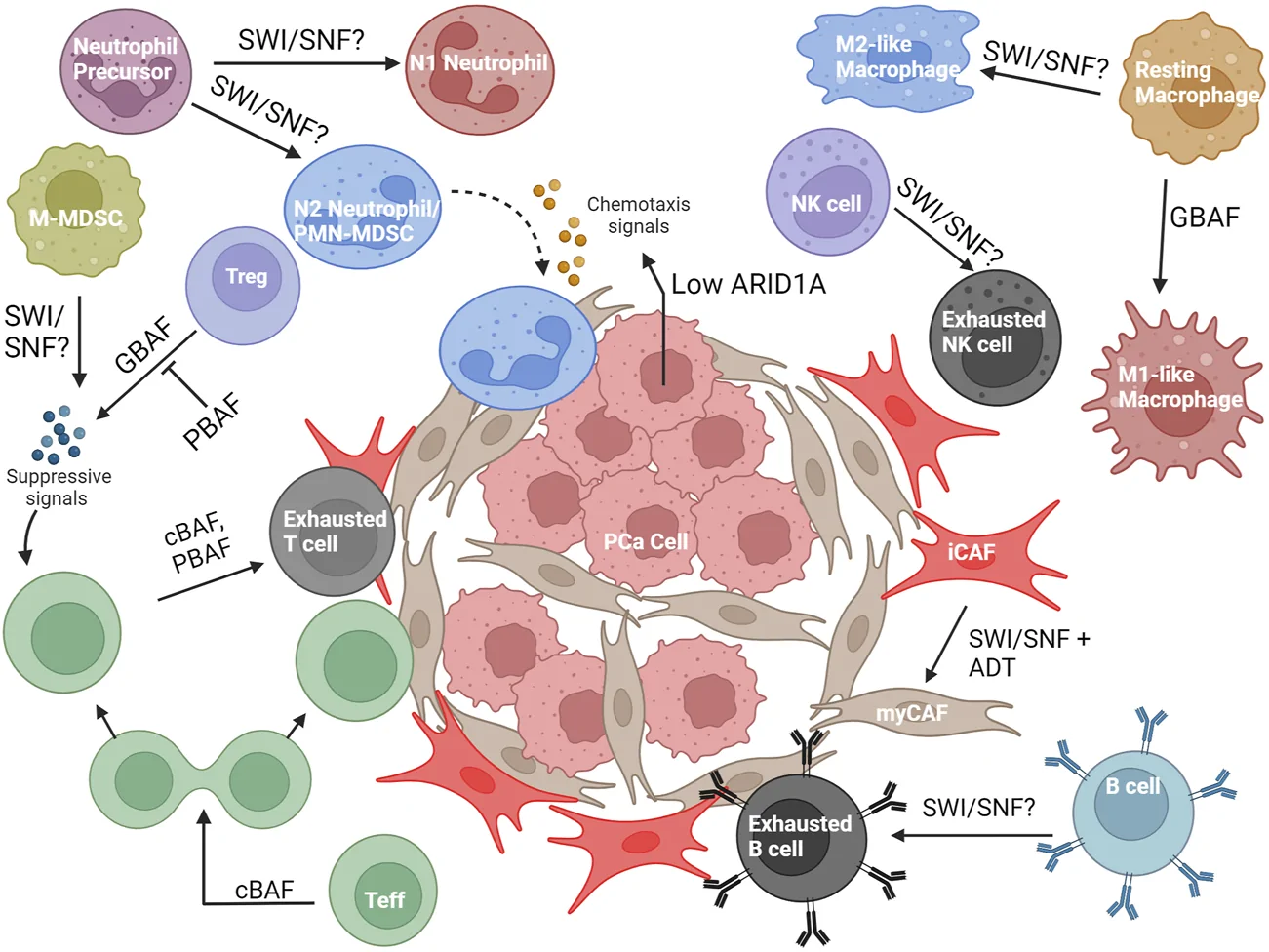
Overview of the known and possible roles SWI/SNF may have in the PCa microenvironment. Abbreviations: PCa, prostate cancer; ADT, androgen- deprivation therapy; Teff, T effector cell; Treg, T regulatory cell; NK cell, Natural killer cell; PMN-MDSC, Polymorphonuclear myeloid-derived suppressor cell; M-MDSC, Mononuclear myeloid-derived suppressor cell; iCAF, inflammatory carcinoma-associated fibroblast; myCAF, myofibroblastic carcinoma- associated fibroblast.

### 5.1 Carcinoma associated fibroblasts (CAFs)

CAFs are a heterogenous group of fibroblasts that have differing and overlapping functions to support the tumor cells and the greater microenvironment (Glabman, Choyke, and Sato, 2022). Although many subtypes of CAFs exist, the two most common are myofibroblastic CAFs (myCAFs) and inflammatory CAFs (iCAFs). myCAFs have high expression of α-smooth muscle actin (α-SMA), low expression of inflammatory cytokines, and are responsive to TGF-β. iCAFs are defined by high IL-6 and IL-11 expression and low α-SMA expression (Glabman et al., 2022). myCAFs support cancer growth by producing an extracellular matrix that protects tumor cells from immune attack and therapeutics, while iCAFs secrete inflammatory cytokines to sequester immune cells from tumor and encourage immune exhaustion (Elyada et al., 2019). While initially discovered in pancreatic cancer (Öhlund et al., 2017; Biffi et al., 2019; Elyada et al., 2019), single-cell RNA sequencing (scRNA-seq) studies have shown these CAF signatures in several other solid tumor cancers (Chen et al., 2020; Wu et al., 2020), including PCa (Wang et al., 2023). iCAF and myCAF fibroblast signatures were identified in genetically-engineered mouse models of PCa with clusters that appear to be transitioning between iCAF and myCAF (Wang et al., 2023), consistent with other studies indicating that CAFs can interconvert (Biffi et al., 2019; Elyada et al., 2019). Blocking androgen signaling in these fibroblasts encouraged conversion to a myCAF-like state, which induce castration resistance through the actions of SPP1 (secreted phosphoprotein 1) (Wang et al., 2023). This CAF conversion was dependent on the transcription factor SOX4; knockdown of *Sox4* in CAFs reduced myCAF-related gene expression and tumor resistance to androgen signaling blockade. In addition, SOX4 associates with SMARCA4, SMARCC1, and ARID1A (via co-IP), with SOX4 and SMARCA4 both binding at the *Spp1* locus (via ChIP-qPCR). In a SOX4 overexpression CAF model, knockdown of *SMARCA4* significantly reduced transcription of myCAF-related genes while not changing iCAF-related gene transcript levels (Wang et al., 2023), suggesting that the interconversion from iCAFs to myCAFs in PCa is dependent on SWI/SNF function. This implicates SWI/SNF as a potential target to inhibit anti-androgen therapy resistance mediated by myCAFs.

### 5.2 Immune cells

There has been very little investigation into the roles of SWI/SNF in the immune cells specifically found in the PCa microenvironment, such as monocytic MDSC (M-MDSC) and polymorphonuclear MDSC (PMN-MDSC). However, in some relevant immune cells, the dependencies on SWI/SNF may be applicable to the PCa microenvironment. We have highlighted the studies performed on immune cell types potentially relevant to the PCa TME below.

#### 5.2.1 Macrophages

In the TME, macrophages can have both a pro-inflammatory (M1-like) or anti-inflammatory (M2-like) role (Palano et al., 2022). M1-like macrophages inhibit tumor growth by recruiting adaptive immune cells, whereas M2-like macrophages promote cancer growth by immune suppression, vascularization of the tumor, and promoting metastasis (Palano et al., 2022). In macrophage development, SWI/SNF complexes are known to control lineage-determining transcription factors (LDTFs) (Gatchalian et al., 2020), as demonstrated by *Arid1a* knockout mice decreasing chromatin accessibility to the LDTFs PU.1, RUNX1, GATA, and CSF-1 (L. Han et al., 2019). Furthermore, LDTFs recruit SWI/SNF subunits to enhancers of macrophage-specific genes, such as PU.1 recruiting SMARCC1 and SMARCB1 to the *Il12b* and *Il1a* loci in mature macrophages (McAndrew et al., 2016). SWI/SNF is recruited to other pro-inflammatory early and late response genes (Ramirez-Carrozzi et al., 2006; Liu et al., 2023), through AKIRIN2 (Tartey et al., 2014), KDM2B (Zhou et al., 2020) or the non-coding RNA transcript from *Cox2* (LincRNA-Cox2) (Hu et al., 2016), and cooperates with p300 to increase expression of DNA repair enzymes that control DNA damage from macrophage activity (Pietrzak et al., 2019).

GBAF specifically has been found to be important for macrophage function. BRD9 is important for inflammatory gene expression in bone marrow-derived macrophages treated with lipopolysaccharide (LPS) and BRD9 inhibitors can enhance the anti-inflammatory response mediated by dexamethasone-induced glucocorticoid receptor activity (Wang et al., 2021). Similarly, bone marrow-derived macrophages require BRD9 for the expression of secondary response genes in the IFN pathway when stimulated with the endotoxin Lipid A via cooperation with the ISGF3 transcription factor complex, which regulates interferon-stimulated genes (Ahmed et al., 2022). Given these findings, SWI/SNF is clearly important for proinflammatory (M1-like) macrophage function, which would support an anti-tumorigenic role for SWI/SNF in PCa; however, it is unknown whether SWI/SNF is also important for anti-inflammatory M2-like macrophage function, which could implicate SWI/SNF in a pro-tumorigenic role. Additionally, SWI/SNF has not been investigated specifically in the context of PCa, where pro-tumorigenic M2-like macrophages are prevalent (Han et al., 2022).

#### 5.2.2 Neutrophils

Neutrophils are granulocytic polymorphonuclear phagocytes that serve a role in the innate immune system (Wang, Zhang, and Gao, 2022). Similar to macrophages, neutrophils can differentiate into both a proinflammatory (N1) and an anti-inflammatory (N2) phenotype (Wang et al., 2022). The N1 neutrophils exert anti-tumorigenic activity by releasing factors that support cytotoxicity, such as reactive oxygen species (ROS), Fas, and ICAM-1, while N2 neutrophils support tumor growth by releasing factors such as MMP9, VEGF, CCL2, and CXCL4 (Wang et al., 2022). For clarification, N2 neutrophils and PMN-MDSCs are considered to be the same cell type by some, but this is currently debated in the field (Antuamwine et al., 2023). In metastatic PCa, an increased neutrophil-lymphocyte ratio was associated with poorer outcomes (Su et al., 2019), implicating neutrophils as a potential therapeutic target; however, very few studies have investigated the role of SWI/SNF in their function, and none have addressed this role in the context of PCa. In development, the differentiation from promyelocytes to neutrophils corresponds with a decrease in PBAF subunit expression and an upregulation of an inactive PHF10 isoform (PHF10Ss) without the tandem PHDs in the C-terminal region (Viryasova et al., 2019). Additionally, SMARCD2 (found in cBAF and PBAF) is required for the binding and activity of CCAAT-enhancer binding protein-epsilon (CEBPɛ), which upregulates the expression of secondary and tertiary granule genes important during neutrophil maturation and granule function (Priam et al., 2017). This corroborates the clinical finding that patients with SMARCD2 mutations are associated with defects in neutrophil function (Witzel et al., 2017; Schim van der Loeff et al., 2021). The role of SWI/SNF in the context of tumor-associated neutrophils has not been explored, and from a therapeutic perspective, it would be important to determine whether SWI/SNF-driven chromatin remodeling plays a role in the differentiation to N1 and N2 phenotypes (Wang et al., 2018).

#### 5.2.3 T cells

As in many cancers, T cells have a prominent role in PCa. CD4^+^ T cells can have an anti-tumorigenic role (Th1), pro-tumorigenic role (Treg), or a mixture of both (Th2, Th17). CD8^+^ T cells are important for an anti-tumor response, but can differentiate to an exhausted state, with reduced cytotoxic function (Wang et al., 2022). Lastly, memory T cell (Tmem) generation is important for immunosurveillance, maintaining an anti-tumor immune response, and facilitating the response to immunotherapies (Liu et al., 2020). SWI/SNF has been extensively studied in T cell development and has numerous dependencies (Wurster and Pazin, 2012), including lineage-specific differentiation (De et al., 2011; F. Zhang and Boothby, 2006, 1; Letimier et al., 2007; Wurster and Pazin, 2008; Lee et al., 2020; Chaiyachati et al., 2013); however, to remain in the cancer context as much as possible, we will focus on SWI/SNF function post-activation in this review and highlight several different roles for SWI/SNF that have recently been identified.

*Treg*: In a genome-wide screen of activated CD4^+^ T regulatory cells (Tregs), expression of *Foxp3*, the master transcription factor of Tregs, was reduced upon loss of GBAF subunits (BRD9, GLTSCR1/1L), and increased upon loss of PBAF subunits (BRD7, ARID2, PBRM1, and PHF10) (Loo et al., 2020). Furthermore, loss of GBAF or PBAF-specific subunits, respectively, decreased and increased suppression activity of Tregs when co-cultured with T effector cells (Teff). Treatment with the molecular degrader dBRD9 was able to recapitulate these effects seen in BRD9 knockout (Loo et al., 2020), highlighting a druggable target that could increase the antitumor immune response in solid tumors.

*Tmem:* Memory T cell (Tmem) formation has also been investigated in two recent major studies (Guo et al., 2022; McDonald et al., 2023) In the first study, a genome-wide CRISPR screen in ovalbumin peptide-specific OT-1 T cells identified an increase in Tmem generation with loss of *Arid1a* and *Smarcd2* prior to activation. It was concluded that upon activation, cBAF and c-Myc are asymmetrically distributed upon T cell division; cells with lower amounts of cBAF and c-Myc became Tmem, and cells with high amounts became Teff. Additionally, it was demonstrated that *ex vivo* treatment with the cBAF inhibitor BD98 in CD8^+^ T cells and in CAR T cells resulted reduced tumor growth in mouse models (A. Guo et al., 2022), suggesting a druggable target in the cancer setting. The second study utilized a lymphocytic choriomeningitis virus (LCMV)-specific CD8^+^ T cell clone with a floxed *Arid1a* allele and *Gzmb-Cre^+^* such that *Arid1a* knockout occurs right at the point of activation instead of prior to activation. With this model, it was found that Teff cell expansion was dependent on *Arid1a* as cBAF was required for maintaining chromatin accessibility for the enhancers of transcription factors important for early activation and effector differentiation, such as *Tbx21* (T-bet) and *Zeb2* (McDonald et al., 2023). Furthermore, this loss of chromatin accessibility at the point of activation effected Tmem cells as well. While Tmem generation was not changed, cytokine production by circulating Tmem cells was impaired and tissue-resident Tmem cells were reduced. (McDonald et al., 2023). To summarize, upon activation, if cBAF has been previously knocked out prior to activation, naïve T cells will prefer maturing to the memory phenotype due to the absence of cBAF. If cBAF is knocked out right at the point of activation, then enhancer regions at the loci of important transcription factors, such as T-bet, are reduced. This causes dysfunctional Tmem activity, reduced Teff population, and reduced tissue-resident Tmem cells (Figure 8A). Both studies showed cBAF was important for differentiation and proliferation of Teff cells (Guo et al., 2022; McDonald et al., 2023). From these results, more questions arise of the exact role of cBAF in T cell activation and how that role changes temporally.

**FIGURE 8:**
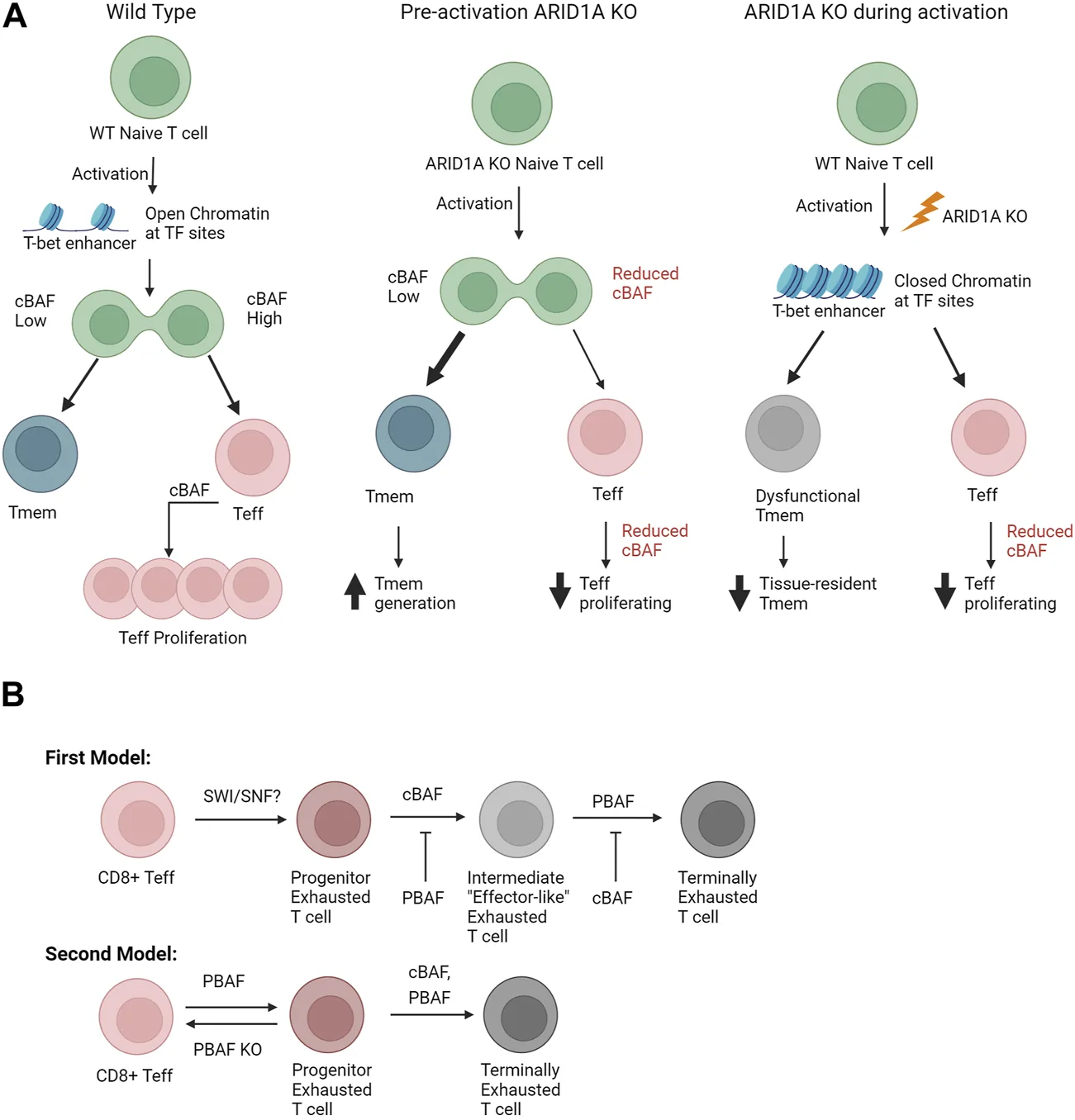
**(A)** Comparison of different modalities to study cBAF in memory T cell formation. The middle pathway is similar to the work performed by (A. Guo et al., 2022). The reduced cBAF before activation leads to a lower amount of Teff proliferation and increased amount of Tmem generation; this is thought to be due to the asymmetric expression of cBAF upon cell division after activation. The right pathway summarizes the work performed by (McDonald et al., 2023). Knocking out ARID1A at the point of activation leads to a decrease in transcription factors important for T cell activation and differentiation, such as Tbx21 (T-bet). This in turn leads to a reduced Teff population and a dysfunctional circulating Tmem population and a decreased tissue-resided Tmem population. **(B)** Suggested exhaustion models of SWI/SNF driven exhaustion. The first model is based on (Baxter et al., 2023). The second model is based on (Kharel et al., 2023). Both models loosely support the findings from (Belk et al., 2022; Battistello et al., 2023), as both articles found cBAF important to reach the terminally exhausted stage. Abbreviations: Teff, T effector cell; Tmem, Memory T cell; WT, wildtype; KO, Knockout.

*Exhausted T cells:* The process of T cell exhaustion is dependent on SWI/SNF as well. CD4^+^ T cells that were exhausted via breast cancer cell coculture had increased SMARCA4 binding to the *CD274* locus and increased PD-L1 expression (Jancewicz et al., 2021). In a genome-wide CRISPR screen of mouse CD8^+^ T cells *in vitro, Arid1a* was required for the transition to the exhausted state, and deletion thereof increased T cell persistence (Belk et al., 2022). Moreover, in solid tumor xenograft experiments, T cell-specific *Arid1a* deletion increased overall survival and decreased tumor burden (Belk et al., 2022). These observations were later corroborated in a separate study where an *in vitro* genome-wide CRISPR screen of overstimulated murine CD8^+^ T cells showed major depletion of *Arid1a* and *Dpf2* knockouts in T cells with high PD-1 and TIM-3, indicating exhaustion (Battistello et al., 2023). Guide RNAs for *Smarcc1, Smarca4, Arid2, Pbrm1,* and *Arid1b* were also depleted, albeit to a lesser extent. Increased T cell persistence was observed in pan-SWI/SNF and cBAF-specific subunit-knockout CD8^+^ T cells after 9 days of activation. Furthermore, pretreatment of CD8^+^ T cell and CAR T cells with inhibitors/degraders of SMARCA4/2 decreased the number of exhausted T cells and increased persistence in solid tumor models as well (Battistello et al., 2023).

Using CD4^CRE+^ Cas9^+^ LCMV infection model, an *in vivo* genome-wide CRISPR screen found PBAF subunits (mainly *Arid2* and *Pbrm1*) important for blocking the transition of early progenitor exhausted state in CD8^+^ T cells to an intermediate exhausted state and inducing the transition of the intermediate to terminally exhausted state (Baxter et al., 2023). Thus, blocking PBAF subunits caused an accumulation of this intermediate exhausted T cell state, which is “effector-like.” It was also found that knocking out *Arid1a* led to less intermediate and more terminally exhausted T cells. Moreover, concomitant loss of PBAF in the T cells improved treatment response in the B16 melanoma model with PD-1 antibody blockade (Baxter et al., 2023). In a separate study, *Arid2* ^fl/fl^ *Vav*Cre mice (*Arid2* conditional deletion in hematopetic cells) infected with LCMV had an increased fraction of Teff and decreased progenitor and terminally exhausted T cells, suggesting that PBAF inhibition can reverse progenitor exhausted T cells back to effector T cells. In agreement, the same effect was observed for T cells in the B16 melanoma model (Kharel et al., 2023). The discrepancy between these studies is intriguing; Baxter et al. essentially showed PBAF inhibits the transformation from a progenitor exhausted state to an intermediate “effector-like” state while promoting the effector-like state to the terminally exhausted state, while Kharel et al. suggested that PBAF promotes the whole effector to exhausted transition (Figure 8B). The discrepancy may be due to the differences in the models. It is also possible the intermediate exhausted population may be more similar to T effector cells than initially thought. Questions still arise, however, on the epigenetic changes occurring during the transition from effector T cells to terminally exhausted T cells and how SWI/SNF controls these changes.

#### 5.2.4 B cells

The biology of tumor-infiltrating B cells is currently a young field; however, there are some established roles in the TME. After activation, B cells can form memory cells or plasma cells (Laumont et al., 2022). Plasma cells typically infiltrate the TME, whereas memory B cells typically stay in lymph tissue or tertiary lymphoid structures. Activated B cells have antibodies designed for epitopes expressed on cancer cells, and thus can contribute to the clearing of these cells with antibody-dependent cellular cytotoxicity (ADCC) and antibody-dependent cellular phagocytosis (Laumont et al., 2022). In PCa, B cells can play a negative role by releasing factors that encourage PCa castration resistance and attenuate the T cell response (Kwon, Bryant, and Parkes, 2021). Exhausted B cells and B “regulatory” cells also exist in the microenvironment, though they are currently not well-studied (Laumont et al., 2022). Similar to T cells, B cells also have SWI/SNF dependencies during development (Morshead et al., 2003; Gao et al., 2009; Osipovich et al., 2009; Choi et al., 2012; Bossen et al., 2015). Despite these findings, little has been studied on SWI/SNF’s role in plasma B cells of the prostate TME.

Many studies have been published about the role SWI/SNF subunits in B cells of germinal centers during activation and differentiation into antibody-producing plasma cells (Holley and Beezly, 2014; Choi et al., 2015; Tartey et al., 2015; Schmiedel et al., 2021). Whether this has an effect in the prostate TME is unknown; although, germinal centers have been implicated as an important part of tertiary lymphoid structures and associated with longer survival in other cancers (Siliņa et al., 2018; Gunderson et al., 2021). Many areas of B cell differentiation are still unexplored. As referenced, memory versus effector formation and exhaustion are currently not studied. How the individual SWI/SNF complexes may be involved is also unanswered.

#### 5.2.5 NK cells

Natural killer (NK) cells are innate cells of lymphoid origin that exhibit cytotoxicity, making them an active area of investigation for anti-cancer immunity (Palano et al., 2022). Upon activation by ligands on cancer cells and through T cell interaction, they release molecules that encourage cancer cell death, including IFNγ, perforin, and granzymes (Palano et al., 2022). Furthermore, they can also eliminate cells tagged with antibodies through ADCC. Similar to T and B cells, NK cells can also become exhausted, reducing anti-cancer efficacy (Palano et al., 2022).

There is little information about SWI/SNF in NK cells. Using murine NK cell lysates, Rautela et al. performed an IP-MS experiment of Inhibitor of DNA-binding 2 (Id2), a protein important for NK cell development by repressing genes that promote T and B cell development, and identified many core SWI/SNF subunits, including SMARCC1, SMARCC2, SMARCA4, ARID1B, SMARCB1, and SMARCD2 (Rautela et al., 2019). Outside of development, a recent study showed exhausted NK cells from oral squamous cell carcinoma downregulated ARID2 in comparison to control splenic NK cells (Li et al., 2023). Knockdown of ARID2 in NK cells reduced pro-inflammatory genes and increased exhaustion markers in comparison to control when injected *in vivo;* overexpression of ARID2 had the opposite effect. However, there was no effect on tumor growth (Li et al., 2023), which may reflect a cancer-dependent role for NK cells. Based on current evidence, it may be possible that PBAF is preventing NK cell exhaustion; however, the role cBAF and GBAF play in this pathway is still unknown, as is SWI/SNF’s role in NK cells on PCa progression and therapy resistance.

### 5.3 Effects of SWI/SNF in cancer cells on the tumor microenvironment

Not only does SWI/SNF affect the function of immune cells in the microenvironment, but its expression within the tumor can affect interaction with the TME. In other cancers, SWI/SNF subunits and subunit mutations/aberrations have been shown to change the composition of the TME and the response to immune therapies (Pan et al., 2018; Li et al., 2020; Miao et al., 2018; Liu et al., 2020). Here we will outline the current knowledge of how SWI/SNF complexes in PCa cells control how the TME is shaped (Hagiwara et al., 2022; Li et al., 2022).

Using data available in the TCGA database and histological analysis of murine PCa tumors, it was discovered that *ARID1A* expression is inversely correlated with tumor aggressiveness in PCa (Li et al., 2022). *ARID1A* loss in *in vitro* organoids of PCa cells did not give a notable growth advantage; however, in an immunocompetent mouse model, PCa cells with *ARID1A* knockout did exhibit a growth advantage due to increased PMN-MDSC recruitment, decreased CD8^+^ T cell infiltration, and decreased IFN-γ expression from the CD8^+^ T cells (Li et al., 2022) (Figure 7). Mechanistically, *ARID1A* deletion reduced accessibility at *A20*, a deubiquitinase that negatively regulates NF-κB signaling, resulting in NF-κB mediated upregulation of *Cxcl2* and *Cxcl3*, cytokines that encourage MDSC recruitment and an immunosuppressive pro-tumor TME. ARID1A is very rarely mutated in PCa but is often downregulated through phosphorylation and proteosomal degradation, representing potential therapeutic strategies to increase the levels of ARID1A to promote an anti-immunogenic immune microenvironment (Li et al., 2022). In a separate study, the interplay between SWI/SNF complexes and MUC1-C and the wound healing response (Kufe, 2020) revealed a role for SWI/SNF in activating IFN- γ signaling in PCa, which is conducive for cancer growth. cBAF (ARID1A) is required to facilitate the transcription of *IFNGR1* (interferon gamma receptor 1) by MUC1-C. MUC1-C, which also activates the expression of PBAF subunits (Hagiwara et al., 2021), cooperates with PBAF to facilitate the expression of IRF1, a downstream transcription factor in the IFN-γ pathway. IRF1 further increases the expression of IDO1, WARS, PTGES, ISG15, and SERPINB9, all of which contribute to immune suppression in the TME (Hagiwara et al., 2022). In summary, this work implicates both cBAF and PBAF to the upregulation of an immune suppressive state.

Many unknowns still exist about whether other effects of SWI/SNF can contribute to PCa to shape the TME. SWI/SNF expression in cancer cells may be controlling pathways that contribute to a more favorable environment for the cancer, such as fibroblast recruitment and blood vessel development. Through increased analysis of patient data and immune competent mice it would be possible to elucidate these effects that may otherwise be hidden in monoculture or immunocompromised mouse models.

## 6 Discussion

PCa is the most frequent cancer in men in the US, with an increasing number of patients eventually becoming resistant to available treatments. Genetic aberrations have been clearly defined depending on the stage of the disease, with mutations or deletions of common tumor suppressors such as *TP53* and *RB1* frequently observed, while transcription factors such as AR, MYC, and FOXA1 are amplified or overexpressed. Due to the highly transcriptional nature of PCa, chromatin regulators such as the SWI/SNF chromatin remodelers are just starting to be explored as therapeutic targets for advanced and therapy-resistant subtypes. In this review we have covered the multiple functions for SWI/SNF in PCa, including different aspects of AR signaling, the relationship between SWI/SNF and other genetic drivers, how SWI/SNF facilitates metastasis and therapy resistance, and finally the role of SWI/SNF in the tumor microenvironment (TME), which plays important roles in disease progression and therapy resistance. Different types of inhibitors and degraders of SWI/SNF subunits have been developed, although very few have been tested in PCa. Degraders of the ATPase subunits are effective in xenograft models; however, it is undetermined whether targeting other subunits may also be an effective and potentially less toxic strategy. Targeting cBAF seems to be most critical for inhibiting AR and transcription factor drivers of PCa, such as TMPRSS2::ERG and FOXA1; however cBAF-selective inhibitors are limited and have not yet been tested in PCa. Meanwhile, GBAF and PBAF inhibitors, while less effective against the core transcriptional circuit in PCa, may be associated with fewer toxic side effects and more useful in resistant and advanced subtypes of PCa, such as the increasingly common TE-NEPC. With additional knowledge regarding the most critical SWI/SNF subunits for viability, resistance, and progression of PCa, SWI/SNF inhibitors may be successfully utilized in combination therapies to increase efficacy and decrease resistance to more conventional therapies. Importantly, by understanding the role of SWI/SNF subunits on both the tumor and the immune microenvironment, SWI/SNF inhibition could be a much-needed mechanism for turning the immunologically ‘cold’ PCa tumors into a “hot” ones, which can improve response to immune checkpoint inhibitor immune checkpoint inhibitor therapy or other immunotherapies. In addition, SWI/SNF inhibition may be effective in targeting immunosuppressive cell types, such as MDSCs and Tregs that are involved in resistance to AR-targeted therapies. Through new genetic studies and the development of novel SWI/SNF inhibitors, the specific SWI/SNF subunits required for different stages of PCa progression and therapy resistance can start to be defined in the appropriate disease-relevant settings.
